# Effect of Different pH Values on the Compressive Strength of Calcium-Enriched Mixture Cement

**Published:** 2014-12-24

**Authors:** Fereshte Sobhnamayan, Safoora Sahebi, Ali Alborzi, Saeed Ghorbani, Nooshin Sadat Shojaee

**Affiliations:** a * Department of Endodontics, Dental School, Shiraz University of Medical Sciences, Shiraz, Iran; *; b * Dental Student, Shiraz University of Medical Sciences, Shiraz, Iran*

**Keywords:** Acid, Alkaline, Compressive Strength, Root Canal Filling Materials

## Abstract

**Introduction:** The aim of this study was to evaluate the compressive strength of calcium-enriched mixture (CEM) cement in contact with acidic, neutral and alkaline pH values. **Methods and Materials:** The cement was mixed according to the manufacturer’s instructions, it was then condensed into fourteen split molds with five 4×6 mm holes. The specimens were randomly divided into 7 groups (*n*=10) and were then exposed to environments with pH values of 4.4, 5.4, 6.4, 7.4, 8.4, 9.4 and 10.4 in an incubator at 37^°^ C for 4 days. After removing the samples from the molds, cement pellets were compressed in a universal testing machine. The exact forces required for breaking of the samples were recorded. The data were analyzed with the Kruskal-Wallis and Dunn tests for individual and pairwise comparisons, respectively. The level of significance was set at 0.05. **Results: **The greatest (48.59±10.36) and the lowest (9.67±3.16) mean compressive strength values were observed after exposure to pH value of 9.4 and 7.4, respectively. Alkaline environment significantly increased the compressive strength of CEM cement compared to the control group. There was no significant difference between the pH values of 9.4 and 10.4 but significant differences were found between pH values of 9.4, 8.4 and 7.4. The acidic environment showed better results than the neutral environment, although the difference was not significant for the pH value of 6.4. Alkaline pH also showed significantly better results than acidic and neutral pH. **Conclusion: **The compressive strength of CEM cement improved in the presence of acidic and alkaline environments but alkaline environment showed the best results.

## Introduction

Calcium-enriched mixture (CEM) cement is a hydrophilic tooth colored cement specially used as a root-end filling material and pulp covering agent because of its excellent biocompatibility and sealing ability [1, 2]. This cement is composed of different calcium compounds like calcium hydroxide, calcium oxide, calcium phosphate, calcium sulfate, calcium silicate and calcium carbonate [2]. CEM cement releases calcium hydroxide (CH) during and after setting [3, 4]. Its antibacterial features is similar to CH and better than mineral trioxide aggregate (MTA) [5]. In comparison with MTA, CEM cement has similar sealing ability and pH, but it has an increased flow rate, decreased working time and film thickness [3, 6]. Cytotoxic effect of this novel cement is similar to MTA and less than intermediate restorative material (IRM) [7, 8]. This cement has excellent bioinductivity as it shows a great capacity to induce hard tissue formation in vital pulp therapy [1, 9].

Asgary *et al.* [10] conducted an animal study and reported its capacity in regenerating periodontal ligament (PDL) and induction of cementogenesis. In different studies this biomaterial has also shown other favorable results in apexogenesis as well as pulpotomy of permanent teeth, management of furcal perforation and internal/external root resorption [11-13]. CEM also has good handling properties and provides an effective seal as root-end filling material [1, 2].

Because of the nature of different endodontic procedures like root-end surgery, perforation repair, apexification, *etc*. reparative materials may be placed in contact with inflamed tissues. The amount of pH value in tissues decreases in the presence of inflammation, abscess or periapical pathosis [14]. Thus choosing an appropriate reparative material which is less or not affected by changes in the environmental pH, is recommended. For MTA, as a widely used root-end filling material, properties like sealing ability, tensile strength, push-out bond strength and surface hardness are affected by acidic environment [15-19]. CEM cement and MTA release CH during and after setting [3, 4]. CH reduces the bacterial contamination [20] and has denaturing effect on proinflamatory mediators [21]. Because of high alkalinity, CH can also neutralize the acidic environment; thus pretreatment with CH paste before MTA placement has been recommended specifically for its antibacterial properties [22]. A recent study on the effects of alkaline environment on MTA showed a decrease in its surface hardness in the pH values of 10.4 and 7.4 [23]. The push-out bond strength of MTA is also reduced in the presence of alkaline pH [24]. Therefore, it is hypothesized that changes in the pH value of host tissues in contact with CEM cement may also change its physical/chemical properties. 

Compressive strength is regarded as one of the most important physical characteristics of root-end filling materials that is correlated to their stage of hydration in hydraulic cements [25]. This entity is the highest vertical compressive load that a material can stand before fracture and is measured by universal testing machine [26]. The purpose of this laboratory study is to evaluate the compressive strength of CEM cement after exposure to a range of acidic and alkaline pH levels.

## Methods and Materials

Fourteen custom made two-part plexi glass moulds were used in this experimental study. Each mould had 5 holes with internal diameter of 4 mm and height of 6 mm. Each hole was filled with CEM cement (BioniqueDent, Tehran, Iran). CEM cement was mixed according to the manufacturer’s instructions and applied incrementally into the moulds with moderate forces. An appropriate condenser was used to condense the mixture and excessive material was removed with wet cotton pellets.

The filled moulds were then randomly allocated to seven groups (*n*=10). Pieces of gauze that had been soaked in butyric acid (BA) buffered to pH values of 4.4, 5.4, and 6.4 were placed in the bottom of each container in groups 1 to 3. the sample of control group (group 4) was put on a piece of gauze soaked in synthetic tissue fluid (STF) that was prepared with 1.7 g of KH_2_PO_4_, 11.8 g of Na_2_HPO_4_, 80.0 g of NaCl and 2.0 g of KCl in 10 L of H_2_O (pH=7.4). In groups 5 to 7 the moulds were placed in pieces of gauze soaked in STF buffered in potassium hydroxide at pH values of 8.4, 9.4, and 10.4, respectively. All the moulds were covered with moist pieces of gauze without close contact to ensure the presence of sufficient humidity inside the container. The containers were sealed and kept in an incubator for 4 days at 37^°^C. The acid-soaked and alkaline-soaked pieces of gauze were refreshed every 24 h to ensure a consistent pH during the experimental period. After 4 days, the samples were solid when probed with an explorer before removal. The CEM cement specimens were then removed from the moulds and inspected visually to ensure they had no voids or flaws before being subjected to the compressive strength test.

**Table 1:** Mean (SD) of compressive strength in different pH values

**pH value**	**Mean (SD)**
**4.4**	24.44 (14.52)
**5.4**	36.14 (18.44)
**6.4**	20.06 (10.48)
**7.4**	9.67 (3.16)
**8.4**	37.91 (5.77)
**9.4 **	48.59 (10.36)
**10.4**	44.72 (6.11)

***Compressive strength test***

The pressure on each specimen was applied by a universal testing machine (Instron Universal Testing Machine, Model TM-M, Instron Corp., Canton, Mass, USA). The samples were placed vertically on the steel plate of the machine towards the calibrated steel cross-head plate at a speed of 1 mm per min. When both plates were in contact with the samples, the compressive load was recorded. This loading failure was used to calculate the compressive strength of CEM cement samples using the following formula: CS=4*p*/*πd*^2^ where CS is the compressive strength, *P* is loading failure in Newton (N) and *d* is the diameter of the samples in mm. The compressive strength of all specimens was recorded in MPa. The mean compressive strength, and standard deviation (SD) of each group were calculated and analyzed using the Kruskal-Wallis test for individual comparisons and Dunn test for pairwise comparisons with the significance level set at 0.05. 

## Results

**Figure 1:**
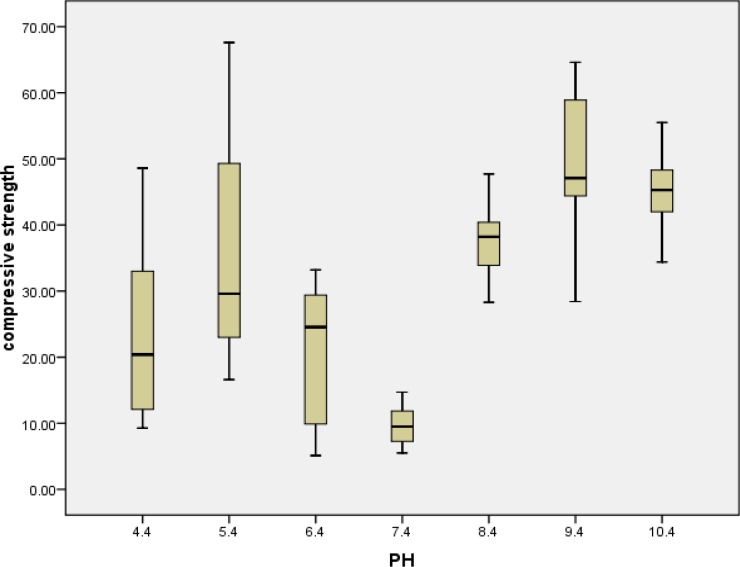
The effect of pH on the compressive strength of CEM cement

The median (Mean±SD) of compressive strengths and standard deviations for experimental groups are shown in Table 1 and Figure 1. The greatest and the lowest mean compressive strength was observed after exposure to pH values of 9.4 and 7.4, respectively. The exact amount of *P*-values and pairwise comparison of the groups are presented in Table 2.

## Discussion

This study was designed to measure the compressive strength of CEM cement in three acidic, neutral and alkaline environmental pH values. The highest and the lowest compressive strength belonged to pH values of 9.4 and 7.4, respectively.

**Table 2:** Pairwise analysis of compressive strength in different pH values (*demonstrates statistically significant differences)

**pH level**		***P*** **-value**
**4.4**	5.4	0.07
	6.4	0.68
	7.4*	0.00
**5.4**	6.4	0.07
	7.4*	0.00
**6.4**	7.4	0.06
**7.4**	8.4*	0.00
	9.4*	0.00
	10.4*	0.00
**8.4**	9.4*	0.01
	10.4*	0.02
**9.4**	10.4	0.31

An ideal endodontic material should be able to tolerate the functional forces during mastication [27-29]. Considering the wide application of endodontic materials such as repair of furcation perforation [30, 31], direct pulp capping [32], vital pulp therapies and placement of coronal restorative material [33], the compressive strength of the used material is an important factor.

In this study STF was used to simulate the neutral and physiological condition of the body [34]. In certain cases such as tissue infection/inflammation, freshly mixed CEM cement is placed in an acidic environment [14]. Here BA was used to simulate the clinical situation of an abscess environment because BA is the metabolic byproduct of anaerobic bacteria [35-37].

Our results showed that not only the acidic environment did not have an adverse effect on the compressive strength of CEM cement but also it improved its compressive strength compared to neutral condition. The difference was significant in all pH values except for 6.4. This finding is not in accordance with the results of some studies on MTA showing that the acidic environment had a negative impact on its setting properties [38], strength [39], hardness and porosity [18, 19], compressive strength, push-out bond strength [15] and sealing ability [19]. This could be related to the high percentage of small-sized particles found in CEM cement that are less affected by the changes in the environmental pH [40]. This can be confirmed with the results of a study on an experimental MTA formulation with nanosized particles that showed better physical and chemical properties in acidic environment [39]. The compressive strength of this formulation was less affected by acidic environment [34]. On the other hand, the faster setting time of CEM cement may cause a shorter working time and a faster chemical reaction which is the most important period for structure formation and ion release [6]. This earlier structure formation and the ion release may cause CEM cement to be less affected by acidic environment. These are just theories and can be a new subject for researchers.

In necrotic immature teeth with previous CH intracanal medication, CEM cement apical plug is placed in an alkaline pH environment. The present study revealed that the greatest compressive strength values were observed in alkaline environment, which was significantly different from neutral and acidic environment. The greatest mean value was seen after exposure to pH of 9.4. This finding is confirmed with the results of another study on some modifications of MTA which showed higher compressive strength in pH value of 10.4 [34]. Two separate studies on the effect of alkaline pH on microhardness [23] and push-out bond strength of MTA [24] showed higher surface hardness in the presence of alkaline pH. They also showed significantly lower surface hardness, higher porosity and more non hydrated structure in neutral condition (pH=7.4) compared with pH values of 8.4 and 9.4 [23]. This finding is partly in accordance with the present study; however, a significant difference between the compressive strength of CEM cement at pH values of 8.4 and 9.4 was observed.

The present study confirms that alkaline pH can increase the strength of root end filling materials. The current study also elucidated that CEM cement kept its high strength in acidic environment (pH values of 4.4 and 5.4) similar to infectious and inflammatory areas. Further studies are needed for evaluation of the porosity and microstructure of this cement when exposed to different pH levels.

## Conclusion

Both acid and alkaline pHs enhanced the compressive strength of CEM cement compared to neutral pH. However, in alkaline pH the compressive strength was still higher than an acidic environment.
